# Disentangling the Cost of Orphan Drugs Marketed in the United States

**DOI:** 10.3390/healthcare11040558

**Published:** 2023-02-13

**Authors:** Hana Althobaiti, Enrique Seoane-Vazquez, Lawrence M. Brown, Marc L. Fleming, Rosa Rodriguez-Monguio

**Affiliations:** 1 Department of Clinical Pharmacy, College of Pharmacy, Umm Al-Qura University, Makkah 24382, Saudi Arabia; 2 Department of Pharmaceutical Economics and Policy Department, Chapman University School of Pharmacy, Irvine, CA 92618, USA; 3 Economic Science Institute, Argyros School of Business and Economics, Chapman University, Orange, CA 92866, USA; 4 Department of Clinical Pharmacy, School of Pharmacy, University of California San Francisco, San Francisco, CA 94143, USA; 5 Medication Outcomes Center, University of California San Francisco, San Francisco, CA 94143, USA; 6 Philip R. Lee Institute for Health Policy Studies at the University of California San Francisco, San Francisco, CA 94143, USA

**Keywords:** orphan drugs, non-orphan drugs, price, market entry

## Abstract

The increasing number and high prices of orphan drugs have triggered concern among patients, payers, and policymakers about the affordability of new drugs approved using the incentives set by the Orphan Drug Act (ODA) of 1983. This study evaluated the factors associated to the differences in the treatment cost of new orphan and non-orphan drugs approved by the FDA from 2017 to 2021. A generalized linear model (GLM) with the Gamma log-link analysis was used to ascertain the association of drug characteristics with the treatment costs of orphan and non-orphan drugs. The results of the study showed that the median and interquartile range (IQR) drug cost was USD 218,872 (IQR = USD 23,105) for orphan drugs and USD 12,798 (IQR = USD 57,940) for non-orphan drugs (*p* < 0.001). Higher market entry prices were associated with biologics (108%; *p* < 0.001), orphan status (177%; *p* < 0.001), US sponsor companies (48%; *p* = 0.035), chronic use (1083%; *p* < 0.001), treatment intent (163%; *p* = 0.004), and indications for oncology (624%; *p* < 0.001) or genetic disorders (624%; *p* < 0.001). Higher market entry treatment cost for newly approved drugs were associated with biologics, orphan status, US sponsor companies, chronic use, therapeutic intent, and indications for oncology or genetic disorders.

## 1. Introduction

In the United States (US), orphan drugs are indicated for the treatment of rare diseases and conditions affecting fewer than 200,000 patients [1]. With an estimated 7000 orphan diseases, 1 out of every 10 Americans live with a rare condition [2]. The Orphan Drug Act (ODA) was introduced in 1983 to encourage the development of new drugs for such conditions. The orphan designation introduced by the Orphan Drug Act of 1983 allows drug manufacturers to benefit from several incentives, such as market exclusivity, fee waivers, direct funding for research and development (R&D), and tax credits that aim to boost returns on investment in orphan drug research and development [3,4].

The Increase in the demand for orphan drugs to address a growing number of rare diseases coupled with the steady increase in prices has raised concerns about the affordability of orphan drugs [5,6,7,8]. New drugs are expensive and contribute to rising healthcare costs for public and private patients [9,10,11], and the FDA orphan designation is associated with higher prices and out-of-pocket expenditures [12,13].

However, studies assessing the factors behind differences in the costs of orphan and non-orphan drugs in the US are lacking. This study evaluated the factors associated to the differences in the treatment cost of new orphan and non-orphan drugs approved by the FDA from 2017 to 2021.

## 2. Material and Methods

### 2.1. Data Sources

We extracted the list of new molecular entities and therapeutic biologics approved and marketed in the US in 2017–2021 from the FDA website [14]. Vaccines, allergenic products, and blood and blood products were excluded from the study. We collected the first wholesale acquisition costs (WACs) from the IBM Micromedex RED BOOK and used the WACs at market entry as proxies for the actual acquisition costs by private payers. Pharmaceutical companies use the WACs to set the initial reference price in the Medicaid outpatient pharmacy, 340B Drug Pricing Program, and Federal Supply Schedule programs [15,16]. The Medicare Part B program also uses the WACs to set the initial prices for reimbursement of drugs used in physician offices. We collected price data at the national drug code (NDC) level and selected the lowest NDC cost per unit at market entry whenever several NDCs were available for the same active ingredient, dosage form, and strength. We selected the unit (tablet, capsule, vial, etc.,) closest to the FDA-recommended strength when a drug had several strengths. We classified the approved drugs in the following therapeutic categories [17]: genetic disorders, HIV and related comorbidities, other infectious diseases, oncology, transplants, and other areas.

We collected each drug’s recommended dose and treatment duration from the first FDA approved label. When the FDA-approved label did not indicate the treatment duration, we used the median treatment duration from pivotal clinical trials listed on the label. We assumed an average patient weight of 70 kg and a body surface area of 1.75 m^2^ to calculate the daily dose for adult patients, and 40 kg was used to calculate the daily dose for pediatric patients if any adjustment was needed (Appendix A, Table A1).

We calculated the treatment cost for single-use, use for less than one year, and use for one year or longer. We inflated the prices to USD 2021 using consumer price index (CPI) non-seasonally adjusted data for all US city average items and all urban consumers from the US Bureau of Labor Statistics [18].

### 2.2. Data Analysis

We conducted descriptive statistics for each variable included in the analysis. Then, we studied the correlations between the treatment cost of newly approved drugs at market entry and the variables, as well as between the variables themselves. We used the Chi-squared test or Fisher’s exact test in combination with the Phi-coefficient or Cramer’s V considered in cases where both variables were categorical. If both variables were continuous, scatter plots were depicted, and Spearman or Bravais–Pearson correlation coefficients were calculated. We used the point–biserial correlation to check for correlations between categorical and continuous variables. Kruskal–Wallis test is also conducted to check for a significant difference between the means of the ordinal variables’ groups (Appendix A, Table A2).

### 2.3. Study Outcome: The Treatment Costs of New Approved Drugs at Market Entry

We used a generalized linear model (GLM) with the Gamma log-link to assess the association between the treatment costs of newly approved drugs at market entry and potential variables: the date of first market entry, application type (New Drug Applications (NDAs), Biologic License Applications (BLAs)), country of incorporation of the sponsor company (US vs. non-US), a binary indicator for first-in-class, a binary indicator for orphan drugs, FDA review type (standard vs. priority), therapeutic intent (diagnosis, prevention, or treatment), therapeutic area (genetic disorders, HIV and related comorbidities, other infectious diseases, oncology, transplants, and other areas), age group (adult, pediatric and adult, or pediatric), and treatment duration (single-use, less than one year, or one year or longer) while addressing the right-skewed distribution of our data. We included all statistically significant variables (*p* < 0.05) from the bivariate analysis in the GLM. We tested for multicollinearity among independent variables in the GLM using the variance inflation factor (Appendix A, Table A3).

We used the train-test split procedure to estimate our model’s performance and prevented the model from overfitting by using root-mean-square error (RMSE). We used two-tailed statistical tests and a *p* value of 0.05 as the significance threshold. We conducted all analyses using RStudio statistical software (version 4.0.3).

## 3. Results

The FDA approved 257 new drugs, including 127 (49.4%) orphan and 130 (50.6%) non-orphan drugs in 2017–2021. We excluded 15 drugs that were not marketed in the US as of March 31, 2022; thus, the analytical sample included 242 drugs, including 118 (48.8%) orphan drugs and 124 (51.2%) non-orphan (Table 1).

The percentages for orphan drugs versus non-orphan drugs were as follows: therapeutic biologics (54.0% vs. 46.8%), US country of incorporation of the sponsor company (50.3% vs. 49.7%), first-in-class (59.2% vs. 40.8%), and intended for treatment (50.2% vs. 49.8%).

Orphan drugs also had higher percentages of approvals for FDA-expedited review processes and other regulatory designations (62.4% vs. 37.6%), priority review designations (63.4% vs. 36.6%), accelerated approvals (73.9% vs. 26.1%), breakthrough therapy designations (74.4% vs. 25.6%), and fast-track designations (59.8% vs. 40.2%). Similarly, orphan drugs accounted for higher percentages of approved new oncology drugs (63.5% vs. 36.5%) and genetic disorder drugs (95.1% vs. 4.9%; Table 1). The most frequently approved new drugs for pediatric patients were orphan drugs (83.3% vs. 16.7%), adult and pediatric drugs (73.7% vs. 26.3%), and adult drugs (40.3% vs. 59.7%; Table 1).

### 3.1. Treatment Cost of New Approved Drugs at Market Entry

**Figure 1:**
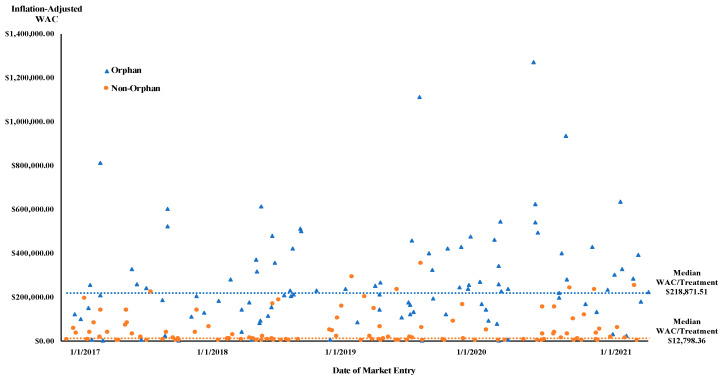
New drugs treatment cost at US market entry (USD 2021) and median WAC.

The median treatment cost was USD 218,872 for orphan drugs (IQR = USD 231,057, range USD 237–USD 1,272,021) and USD 12,798 for non-orphan drugs (IQR = USD 57,940, range USD 44–USD 382,866, *p* < 0.001; Figure 1; Appendix A, Table A1).

Compared with non-orphan biologics drugs, the median treatment cost was 4.3 times higher for orphan therapeutic biologics (USD 264,007.88 vs. USD 61,468.75, *p* < 0.001) and 3.2 times higher for orphan fixed drug combinations (USD 100,177.88 vs. USD 30,895.32, *p* < 0.001; Table 1).

**Figure A1:**
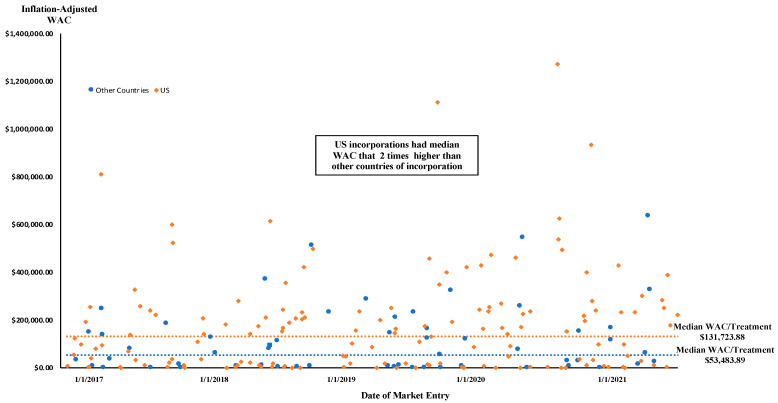
New drugs treatment cost at US market entry (USD 2021) and median WAC.

The median treatment cost was higher for orphan drugs marketed by US companies than for companies from other countries (USD 237,265 vs. USD 128,580, *p* = 0.005; Table 1; Appendix A, Figure A1).

However, the difference in the median treatment cost for non-orphan drugs marketed by US companies and those marketed by companies from other countries was insignificant (USD 9483.82 vs. USD 15,834.14, *p* = 0.262; Table 1).

The median treatment cost for first-in-class approved orphan drugs was not statistically significant relative to the median treatment cost for other orphan drugs (USD 239,593.23 vs. USD 206,176.28, *p* = 0.322). The median treatment cost for orphan drugs that received a priority review was not significantly different from the cost for orphan drugs with standard reviews (USD 233,934.14 vs. USD 142,195.27, *p* = 0.053).

Although orphan drugs intended for treatment had a median treatment cost three times higher than drugs for preventive use, the difference was not statistically significant (USD 230,768.11 vs. USD 71,503.98, *p* = 0.190). The median treatment cost for non-orphan drugs intended for treatment indication was significantly higher than for drugs for the preventive indication (USD 18,486.88 vs. USD 2311.92, *p* = 0.047).

**Figure A2:**
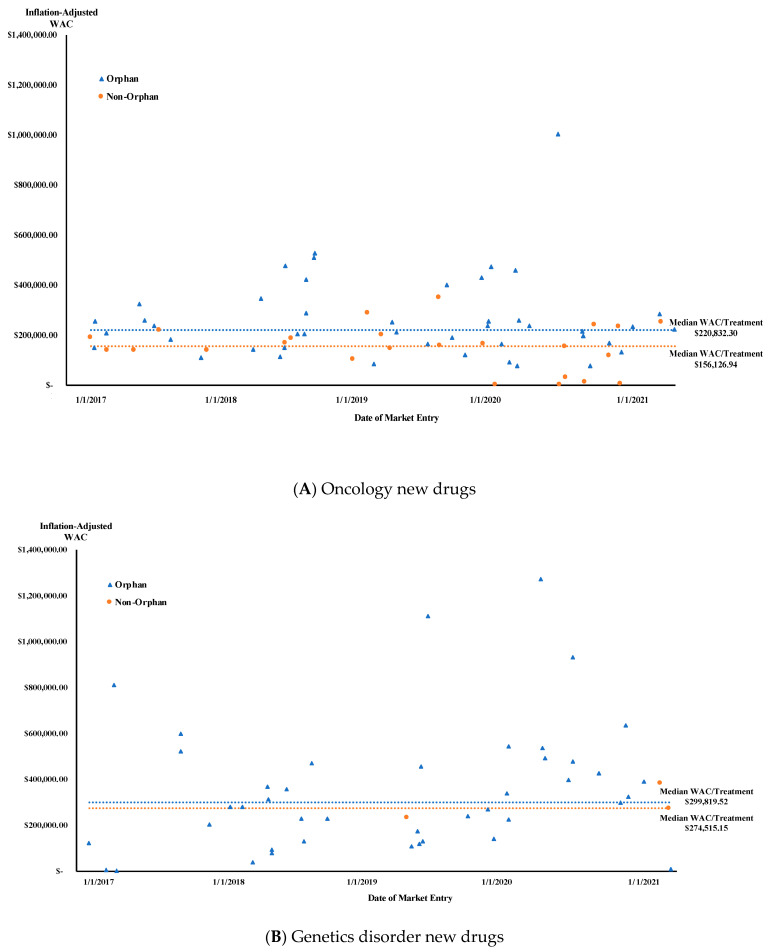
New drugs treatment cost at US market entry (USD 2021) and median WAC.

For the therapeutic areas, we identified a significant difference in the median treatment cost for oncology orphan drugs compared to non-orphan drugs (USD 220,832.30 vs. USD 156,126.94, *p* = 0.002; Appendix A, Figure A2). Finally, the median treatment cost across patient age groups was significantly higher for orphan drugs targeting both adult and pediatric populations than for non-orphan drugs (USD 280,152.74 vs. USD 1067.40, *p* < 0.001).

### 3.2. Factors Explaining Treatment Cost of New Approved Drugs at Market Entry

The date of market entry, priority review, and approval as first-in-class drugs were not statistically significantly associated with the mean treatment cost for newly approved drugs at market entry. However, the mean treatment cost at market entry was positively associated with biologics (110%; *p* < 0.001) and orphan drugs (177%; *p* < 0.001). Higher market entry treatment costs were also associated with drugs sponsored by US pharmaceutical companies (67%; *p* = 0.035), drugs intended for treatment rather than prevention (163%; *p* = 0.004), and treatments with a duration of one year or longer compared to single use (1092%; *p* < 0.001; Table 2).

Among the therapeutic areas, the higher market entry treatment costs were significantly associated with drugs indicated for oncology (698%; *p* < 0.001) and genetic disorders (608%; *p* < 0.001) compared to infectious diseases (Table 2). An RMSE was obtained for each train model and test model (RMSE-train model = 3.0 ≈ REMS-test model = 2.8), indicating a good model fit.

## 4. Discussion

This novel study assessing US treatment costs of newly approved drugs at market entry from 2017 to 2021 found that the median treatment cost was 17 times higher for orphan than non-orphan drugs. However, after controlling for the characteristics of the drug, date of market entry, therapeutic class, FDA review designation, country of the sponsor company, therapeutic intent, and treatment duration, the treatment drug cost was 2.8 times higher for orphan than non-orphan drugs.

The median treatment costs for orphan drugs exceeded USD 200,000 at market entry. Over the past 20 years, drug expenditures in the US market have increasingly shifted toward drugs that treat relatively few people [7], and the rapid growth of orphan drug approvals has raised concerns about their pricing and affordability [5]. The high costs of orphan drugs are also associated with large out-of-pocket expenditures [12,13].

The findings that the cost of new drugs is associated with orphan drugs, therapeutic biologics, therapeutic class, therapeutic intent, and long treatment duration align with prior research that found that launch prices of new drugs in the US increased faster for biologics and drugs treating rare diseases [11]. In fact, the financial burden on patients and healthcare payers results in high profits for pharmaceutical companies in marketing orphan drugs, even for a small patient populations [12,19,20].

Previous studies have pointed out that drug development is less costly for orphan than for non-orphan drugs due to smaller and fewer efficacy and safety trials, shorter FDA review time, higher marketing approval success rates, and lower marketing prices [6,12,13,21]. Since rare diseases are often serious or life-threatening, most orphan drugs qualify for designations and regulatory pathways established by Congress to expedite new drug development and FDA review and approvals [22,23]. Our study confirmed that a higher percentage of orphan than non-orphan drugs benefited from FDA-expedited designations and approval pathways.

The pharmaceutical industry has been criticized for high prices and profits from orphan drug incentives in situations that do not meet the Orphan Drug Act’s original intent [24,25]. Orphan designations for marketed drugs and the division of diseases into sub-types to apply for multiple orphan designations have also been associated with delays in generics entry [26]. Moreover, results showed that drugs sponsored by US pharmaceutical companies were significantly associated with higher drug treatment costs at market entry than non-US pharmaceutical companies. The differences in prices of drugs at market entry between US and non-US pharmaceutical companies could be explained by different factors than the country of the sponsored company, such as disease severity, additional non-orphan indications, or route of administration.

Previous studies found that the year of market entry was associated with increased drug prices at US market entry [11,27]. However, our analysis showed no statistically significant association between the date of market entry and treatment drug cost, possibly due to the relatively short period evaluated in our study.

To mitigate the high price of drugs for vulnerable populations, Congress created the 340B program in 1992 that requires pharmaceutical manufacturers to provide front-end discounts (typically 30–50%) for outpatient prescription medicines that serve high numbers of uninsured and poor patients [28]. More than 40% of hospitals in the US are eligible to participate in the 340B program [29]. In 2010, The Affordable Care Act (ACA) of 2010 excluded all sales of drugs that obtained orphan drug approval from the discounts offered by the 340B program to safety-net healthcare providers [30]. Manufacturers of frequently utilized drugs, such as the best-selling drug adalimumab, may identify a new use that meets the definition of a rare disease and obtain FDA approval for an orphan drug indication, thus effectively ceasing the provision of 340B discounts for sales of the drug [31,32].

This study evaluated the cost of drug treatment but not the effectiveness of new drugs approved in the US. However, coverage and reimbursement decisions consider both factors (cost and effectiveness). From an economic perspective, orphan drugs should be subject to the same clinical effectiveness, cost-effectiveness, and budget impact analyses as non-orphan drugs [33,34]. However, healthcare organizations and insurers use special criteria when making orphan drug reimbursement decisions [33]. The economic incentive and ethical imperatives remain unresolved for ensuring access to safe, effective, and affordable treatments for patients with rare diseases [35]. Moreover, balancing the economic incentives to develop and market orphan drugs against the overall benefits and improvements in health outcomes remains critically important [36,37].

A potential reason explaining the high cost of orphan drugs is that R&D expenses for orphan drugs must be recouped from a small number of patients, resulting in high drug treatment costs per patient [21].

A previous study found that the prices of an orphan drugs in Europe were higher for conditions with low prevalence [38]. However, another study concluded that the prices of orphan drugs in the US are unlikely to be driven by the prevalence of the target disease [39]. Further studies are needed to associate disease prevalence and drug treatment costs.

The Orphan Drug Act, enacted 40 years ago, has been credited to have an important role in the development and approval of drugs for rare diseases [36]. As a result, there has been a substantial increase in the number of orphan drugs approved by the FDA, providing therapeutic options for patients with unmet medical needs. However, the high cost of these drugs creates significant financial barriers to patient access and highlights the need for a more sustainable and equitable pricing structure to ensure patients’ access to affordable treatments.

## 5. Limitations

This study used the wholesale acquisition cost (WAC) at market entry as a proxy of the actual acquisition cost by private payers. Companies typically use the WAC to set the initial reference price in the Medicaid outpatient pharmacy, the 340B Drug Pricing Program, and the Federal Supply Schedule programs. As price increases in those programs are limited by the rise in the consumer price index, pharmaceutical companies do not have incentives to reduce the market entry price below the WAC. The Medicare Part B program also used the WAC to set new drugs’ initial price. Public and private payers also use the WAC to estimate drug product reimbursement to pharmacies and providers.

The sample included new molecular entities and new therapeutic biologics approved by the FDA in 2017–2021. The study excluded non-therapeutic biologics and approvals of already marketed drugs. However, these exclusions do not affect the validity of the results for the stated period, although future studies on more extensive and inclusive datasets could further extend the validity of our findings. We also did not consider the potential number of users for each drug. Follow-up studies could evaluate the effect of patient population size on drug prices. We used median treatment costs for the FDA-recommended dose and treatment duration. Future studies could use doses and treatment durations observed in clinical practices and average prices weighted by the number of users to better estimate the societal impact of high-cost drugs.

## 6. Conclusions

Orphan drugs were priced significantly higher than non-orphan drugs at market entry. Higher market entry treatment costs were associated with biologics, orphan status, US sponsor companies, chronic use, therapeutic intent, and indications for oncology or genetic disorders. Future research should assess whether the clinical benefits of orphan drugs justify their high costs.

## Figures and Tables

**Table 1:** Characteristics and median cost of new drugs and biological products approved by the US Food and Drug Administration, 2017–2021.

Drugs Characteristics	Non-Orphan			Orphan		
	No. (%)	Median Cost (USD 2021)	*p* Value	No. (%)	Median Cost (USD 2021)	*p* Value
Total	124 (51.2%)	$12,798.36		118 (48.8%)	$218,871.51	
Application type						
NDA	95 (53.1%)	$8701.27	<0.001	84 (46.9%)	$206,176.28	0.051
BLA	29 (46.8%)	$61,468.75		34 (54.0%)	$264,007.88	
Combination						
Fixed-dose combination	12 (85.7%)	$30,895.32	0.010	2 (14.3%)	$100,177.88	<0.001
Single active ingredient	112 (49.1%)	$12,111.33		116 (50.9%)	$223,076.48	
Country of Incorporation						
US	88 (49.7%)	$15,834.14	0.666	89 (50.3%)	$237,264.66	0.005
Other Countries	36 (55.4%)	$9483.82		29 (44.6%)	$128,579.61	
First in class						
Yes	40 (40.8%)	$19,252.87	0.041	58 (59.2%)	$239,593.23	0.322
No	84 (58.3%)	$9483.82		60 (41.7%)	$206,176.28	
FDA review						
Priority review	56 (36.6%)	$29,093.35	0.003	97 (63.4%)	$233,934.14	0.053
Standard review	68 (76.4%)	$7383.70		21 (23.6%)	$142,195.27	
FDA Designations and Pathways						
Accelerated approval	12 (26.1%)	$163,239.32	<0.001	34 (73.9%)	$209,306.88	0.656
Breakthrough therapy	20 (25.6%)	$102,425.22	0.002	58 (74.4%)	$242,091.05	0.073
Fast track	35 (40.2%)	$28,677.06	0.325	52 (59.8%)	$232,237.26	0.667
Therapeutic Intent						
Diagnosis	4 (57.1%)	$1274.04	0.123	3 (42.9%)	$2527.44	0.085
Prevention	11 (64.7%)	$2311.92		6 (35.3%)	$71,503.98	
Treatment	108 (49.8%)	$18,486.88		109 (50.2%)	$230,768.11	
Therapeutic Area						
Genetic disorders	2 (4.9%)	$290,279.77	<0.001	39 (95.1%)	$274,515.15	0.002
HIV	4 (80.0%)	$37,825.76		1 (20.0%)	$36,982.36	
Infectious diseases	17 (77.3%)	$3152.25		5 (22.7%)	$3207.95	
Oncology	27 (36.5%)	$199,370.90		47 (63.5%)	$156,126.94	
Transplant	0 (0.0%)	$0.00		2 (100.0%)	$25,790.23	
Other	74 (75.5%)	$9557.37		24 (24.5%)	$8411.00	
Age Group						
Adult	111 (59.7%)	$77,064.00	0.019	75 (40.3%)	$15,834.14	<0.001
Pediatric/Adult	10 (26.3%)	$212,437.49		28 (73.7%)	$1067.40	
Pediatrics	3 (16.7%)	$211,046.32		15 (83.3%)	$35,684.60	
Treatment Duration						
Single use	15 (75.0%)	$727.85	0.011	5 (25.0%)	$715.47	0.001
Less than one year	43 (52.4%)	$92,438.35		39 (47.6%)	$12,069.44	
One year or longer	65 (46.8%)	$130,151.75		74 (53.2%)	$23,174.91	

BLA, biologics license application; NDA, new drug application.

**Table 2:** Factors explaining the treatment cost of new drugs at market entry, 2017–2021.

Independent Variable	Treatment Cost of New Drugs at Market Entry		
	Ratio of Means ^a^	95% CI	*p* Value
Date of Market Entry	1.02	0.91–1.15	0.722
Application Type (Reference: NDA)			
BLA	2.10	1.39–3.24	<0.001
Orphan drug (Reference: non-orphan)			
Yes	2.77	1.85–4.17	<0.001
Country of Incorporation (Reference: US)			
Other Countries	−0.67	0.46–0.99	0.035
FDA Regulatory Review Approval Pathway (Reference: Standard review)			
Priority review	1.34	0.86–2.07	0.188
First in Class Drugs (Reference: non-First in class drug)			
Yes	1.28	0.88–1.87	0.187
Intent (Reference: Treatment)			
Diagnosis	−0.30	0.09–1.12	**0**.049
Prevention	−0.39	0.21–0.80	**0**.005
Therapeutic Area (Reference: Infectious diseases)			
Genetic disorders	7.08	2.86–17.15	<0.001
HIV	2.48	0.74–10.89	0.164
Oncology	7.98	3.81–16.22	<0.001
Transplant	1.05	0.21–12.55	0.961
Other	2.06	0.95–4.29	**0**.047
Patient Population Indication (Reference: Pediatrics)			
Adults	−0.86	0.38–1.79	0.653
Pediatric/Adult	−0.73	0.33–1.54	0.402
Treatment Duration (Reference: one year or longer)			
Less than 1 year	−0.77	0.51–1.18	0.189
Single use	−0.08	0.04–0.20	<0.001

a: exponentiated coefficients, a percentage increase in the mean treatment drug cost per unit increase in the covariate. BLA, biologics license application; NDA, new drug application.

## Data Availability

The data used to support the findings of this study will be available upon request.

## Appendix A

**Table A1:** The treatment costs of new molecular entities and new biologics at market entry, 2017–2021.

Product Name (Non-Proprietary Name)	Approval Date	Therapeutic Area	Posology Type	Age Group	Units/Treatment	Adjusted WAC Cost Per Year/Treatment at Market Entry
Non-Orphan Drugs New Therapeutic Biologics						
aducanumab-avwa	7 Jun 2021	Other	Chronic Use (1 year)	Adult	9125 mg	$52,540.51
amivantamab-vmjw	21 May 2021	Oncology	Cycles	Adult	26,985 mg	$233,668.03
anifrolumab-fnia	30 Jul 2021	Other	Cycles	Adult	3900 mg	$60,644.32
benralizumab	14 Nov 2017	Other	Chronic Use (1 year)	Pediatric	195 mg	$35,684.60
brodalumab	15 Feb 2017	Other	Cycles	Adult	5460 mg	$33,131.85
brolucizumab-dbll	7 Oct 2019	Genetic Disorders	Chronic Use (1 year)	Adult	39 mg	$233,934.14
cemiplimab-rwlc	28 Sep 2018	Oncology	Cycles	Adult	6067 mg	$168,790.78
dasiglucagon	22 Mar 2021	Other	Single-Use	Adult & Pediatric	0.6 mg	$522.21
dostarlimab-gxly	22 Apr 2021	Oncology	Cycles	Adult	8667 mg	$117,686.30
dupilumab	28 Mar 2017	Other	Short treatment course	Adult	2400 mg	$6217.48
durvalumab	1 May 2017	Oncology	Cycles	Adult	18,200 mg	$138,311.22
efgartigimod alfa-fcab	17 Dec 2021	Genetic Disorders	Cycles	Adult	18,200 mg	$274,515.15
enfortumab vedotin-ejfv	18 Dec 2019	Oncology	Cycles	Adult	3150 mg	$350,189.92
eptinezumab-jjmr	21 Feb 2020	Other	Cycles	Adult	400 mg	$6148.56
erenumab-aooe	17 May 2018	Other	Chronic Use (1 year)	Adult	840 mg	$7383.70
fam-trastuzumab deruxtecan-nxki	20 Dec 2019	Oncology	Cycles	Adult	6552 mg	$158,518.55
fremanezumab-vfrm	14 Sep 2018	Other	Chronic Use (1 year)	Adult	2700 mg	$4922.47
galcanezumab-gnlm	27 Sep 2018	Other	Chronic Use (1 year)	Adult	1440 mg	$7383.70
guselkumab	13 Jul 2017	Other	Cycles	Adult	650 mg	$68,753.32
margetuximab (anti-HER2 mAb)	16 Dec 2020	Oncology	Cycles	Adult	18,249 mg	$153,735.33
ocrelizumab	28 Mar 2017	Other	Cycles	Adult	1200 mg	$70,996.82
risankizumab-rzaa	23 Apr 2019	Other	Cycles	Adult	750 mg	$155,429.21
romosozumab-aqqg	9 Apr 2019	Other	Short treatment course	Adult	2520 mg	$19,724.18
sacituzumab govitecan-hziy	22 Apr 2020	Oncology	Cycles	Adult	14,200 mg	$163,239.32
sarilumab	22 May 2017	Other	Chronic Use (1 year)	Adult	5200 mg	$37,366.74
tezepelumab-ekko	27 Dec 2021	Other	Chronic Use (1 year)	Adult & Pediatric	2730 mg	$23,808.22
tildrakizumab-asmn	20 Mar 2018	Other	Cycles	Adult	433 mg	$61,468.75
tisotumab vedotin-tftv	20 Sep 2021	Oncology	Cycles	Adult	1680 mg	$251,117.72
tralokinumab-ldrm	27 Dec 2021	Other	Chronic Use (1 year)	Adult	3000 mg	$16,121.70
New Molecular Entities						
abaloparatide	28 Apr 2017	Other	Chronic Use (1 year)	Adult	28,800 mg	$16,383.88
abemaciclib	28 Sep 2017	Oncology	Chronic Use (1 year)	Adult	103,200 mg	$220,369.75
air polymer-type A	7 Nov 2019	Other	Single-Use	Adult	217.4 mg	$740.22
alpelisib	24 May 2019	Oncology	Cycles	Adult	99,000 mg	$288,748.34
amisulpride	26 Feb 2020	Other	Single-use	Adult	5 mg	$43.70
angiotensin ii	21 Dec 2017	Other	Single use	Adult	1 mg	$1605.15
apalutamide	14 Feb 2018	Oncology	Chronic Use (1 year)	Adult	86,400 mg	$140,226.19
atogepant	28 Sep 2021	Other	Chronic Use (1 year)	Adult	3600 mg	$2009.75
avatrombopag	21 May 2018	Other	Short treatment course	Adult	200 mg	$9630.92
baloxavir marboxil	24 Oct 2018	Infectious disease	Single-Use	Adult & Pediatric	80 mg	$165.33
baricitinib	31 May 2018	Other	Chronic Use (1 year)	Adult	720 mg	$26,384.87
bempedoic acid	21 Feb 2020	Other	Chronic use (1 year)	Adult	64,800 mg	$3392.97
betrixaban	23 Jun 2017	Other	Short treatment course	Adult	3360 mg	$688.12
bictegravir, embitcitabine, tenofovir alafenamide	7 Feb 2018	HIV	Chronic Use (1 year)	Adult	18,000 mg	$37,825.76
bremelanotide	21 Jun 2019	Other	Single-use	Adult	2 mg	$759.66
brexanolone	19 Mar 2019	Other	Short treatment course	Adult	126,000 mg	$49,458.10
cabotegravir and rilpivirine	21 Jan 2021	HIV	Chronic Use (1 year)	Adult	7200 mg	$36,138.96
cefiderocol	14 Nov 2019	Infectious disease	Short treatment course	Adult	84 mg	$15,834.14
cenobamate	21 Nov 2019	Other	Chronic Use (1 year)	Adult	72,000 mg	$12,153.21
clascoterone	26 Aug 2020	Other	Short treatment course	Adult & Pediatric	180 mg	$1394.25
darolutamide	30 Jul 2019	Oncology	Chronic Use (1 year)	Adult	432,000 mg	$146,050.77
delafloxacin	19 Jun 2017	Infectious disease	Short treatment course	Adult	12,600 mg	$3096.55
difelikefalin	23 Aug 2021	Other	Chronic Use (1 year)	Adult	5475 mg	$12,164.38
doravirine	30 Aug 2018	HIV	Chronic Use (1 year)	Adult	36,000 mg	$17,720.89
drospirenone and estetrol	15 Apr 2021	Other	Chronic Use (1 year)	Adult	365 mg	$2311.92
elagolix sodium	23 Jul 2018	Other	Chronic Use (1 year)	Adult	54,000 mg	$11,624.06
eravacycline	27 Aug 2018	Infectious disease	Short treatment course	Adult	1960 mg	$1835.23
erdafitinib	12 Apr 2019	Oncology	Cycles	Adult	1080 mg	$102,425.22
ertugliflozin	19 Dec 2017	Other	Chronic Use (1 year)	Adult	5400 mg	$10,332.05
etelcalcetide	7 Feb 2017	Other	Chronic Use (1 year)	Adult	780 mg	$55,718.30
ferric maltol	25 Jul 2019	Other	Chronic use (1 year)	Adult	21,600 mg	$6084.00
finerenone	9 Jul 2021	Other	Chronic Use (1 year)	Adult	7300 mg	$14,041.97
flortaucipir F18	28 May 2020	Oncology	Single use	Adult	1.5ml	$1407.33
fosnetupitant and palonosetron	19 Apr 2018	Other	Single-use	Adult	235 mg	$545.75
fostemsavir	2 Jul 2020	HIV	Chronic Use (1 year)	Adult	432,000 mg	$94,387.79
Gallium 68 PSMA-11	1 Dec 2020	Oncology	Single-use	Adult	6 mCi	$1140.75
glecaprevir and pibrentasvir	3 Aug 2017	Infectious disease	Short treatment course	Adult	18,000 mg	$30,895.32
ibrexafungerp	1 Jun 2021	Infectious disease	Single-Use	Adult & Pediatric	600 mg	$481.65
imipenem, cilastatin, relebactam	16 Jul 2019	Infectious disease	Short treatment course	Adult	70 mg	$19,252.87
istradefylline	27 Aug 2019	Other	Chronic Use (1 year)	Adult	7200 mg	$9483.82
lasmiditan	11 Oct 2019	Other	Single-Use	Adult	50 mg	$715.47
latanoprostene bunod	2 Nov 2017	Other	Chronic Use (1 year)	Adult	360 mg	$3774.85
lefamulin	19 Aug 2019	Infectious disease	Short treatment course	Adult	6000 mg	$1448.92
lemborexant	20 Dec 2019	Other	Chronic Use (1 year)	Adult	1800 mg	$1696.51
lofexidine hydrochloride	16 May 2018	Other	Short treatment course	Adult	30 mg	$3719.59
lumateperone	20 Dec 2019	Other	Chronic Use (1 year)	Adult	15,120 mg	$16,286.54
lusutrombopag	31 Jul 2018	Other	Chronic Use (1 year)	Adult	21 mg	$9095.87
meropenem and vaborbactam	29 Aug 2017	Infectious disease	Short treatment course	Adult	84 mg	$14,831.62
naldemedine	23 Mar 2017	Other	Chronic Use (1 year)	Adult	72 mg	$4541.48
neratinib maleate	17 Jul 2017	Oncology	Cycles	Adult	86,400 mg	$137,624.60
netarsudil	18 Dec 2017	Other	Chronic Use (1 year)	Adult	360 mg	$3528.77
olanzapine and samidor- phan	28 May 2021	Other	Chronic use (1 year)	Adult	3650 mg	$34,296.86
oliceridine	7 Aug 2020	Other	Short treatment course	Adult	27 mg	$485.82
omadacycline	2 Oct 2018	Infectious disease	Short treatment course	Adult	4200 mg	$5917.66
opicapone	24 Apr 2020	Other	Chronic Use (1 year)	Adult	18,000 mg	$7279.58
ozanimod	25 Mar 2020	Other	Chronic Use (1 year)	Adult	331 mg	$87,213.00
ozenoxacin	11 Dec 2017	Infectious disease	Short treatment course	Adult & Pediatric	50 mg	$524.40
piflufolastat f 18	26 May 2021	Oncology	Single-use	Adult	9 mCi	$4498.92
plazomicin	25 Jun 2018	infectious disease	Short treatment course	Adult	7350 mg	$4955.11
plecanatide	19 Jan 2017	Other	Chronic Use (1 year)	Adult	1080 mg	$5385.55
ponesimod	18 Mar 2021	Other	Chronic Use (1 year)	Adult	7300 mg	$99,807.53
pretomanid	14 Aug 2019	Infectious disease	Short treatment course	Adult	35,400 mg	$3689.29
prucalopride succinate	14 Dec 2018	Other	Chronic use (1 year)	Adult	720 mg	$5360.25
relugolix	18 Dec 2020	Oncology	Chronic Use (1 year)	Adult	44,040 mg	$29,093.35
remdesivir	22 Oct 2020	Infectious disease	Short treatment course	Adult & Pediatric	600 mg	$3207.95
remimazolam	2 Jul 2020	Other	Single Use	Adult	5 mg	$395.46
revefenacin	9 Nov 2018	Other	Chronic Use (1 year)	Adult	63,000 mg	$4408.82
ribociclib	13 Mar 2017	Oncology	Cycles	Adult	168,000 mg	$191,363.72
rifamycin SV MMX	16 Nov 2018	Infectious disease	Short treatment course	Adult	2328 mg	$154.09
rimegepant	27 Feb 2020	Other	Short treatment course	Adult	1125 mg	$1638.67
safinamide	21 Mar 2017	Other	Chronic Use (1 year)	Adult	18,000 mg	$4390.22
sarecycline	1 Oct 2018	Other	Short treatment course	Adult & Pediatric	9000 mg	$2718.69
secnidazole	15 Sep 2017	Infectious disease	Short treatment course	Adult	2 gm/1 packet	$284.94
segesterone acetate and ethinyl estradiol	10 Aug 2018	Other	Single-use	Adult	0.013 mg	$2107.51
selinexor	3 Jul 2019	Oncology	Cycles	Adult	5504 mg	$199,370.90
semaglutide	5 Dec 2017	Other	Chronic Use (1 year)	Adult	52 mg	$12,798.36
serdexmethylphenidate and dexmethylphenidate	2 Mar 2021	Other	Chronic Use (1 year)	Adult & Pediatric	18,828 mg	$31,574.56
siponimod	26 Mar 2019	Other	Chronic Use (1 year)	Adult	363 mg	$46,405.16
sodium zirconium cyclosilicate	18 May 2018	Other	Chronic Use (1 year)	Adult	3600 mg	$8411.00
sofosbuvir, velpatasvir and voxilaprevir	18 Jul 2017	Infectious disease	Short treatment course	Adult	33,600 mg	$81,657.26
talazoparib	16 Oct 2018	Oncology	Cycles	Adult	360 mg	$187,225.07
tenapanor	12 Sep 2019	Other	Chronic Use (1 year)	Adult	36,000 mg	$17,331.04
tirbanibulin	14 Dec 2020	Other	Short treatment course	Adult	5 mg	$1003.86
tivozanib	10 Mar 2021	Oncology	Cycles	Adult	276 mg	$239,272.43
trifarotene	4 Oct 2019	Other	Short treatment course	Adult & Pediatric	45 gm	$740.55
trilaciclib	12 Feb 2021	Oncology	Cycles	Adult	2520 mg	$12,069.44
ubrogepant	23 Dec 2019	Other	Single-use	Adult	50 mg	$87.40
upadacitinib	16 Aug 2019	Other	Chronic Use (1 year)	Adult	5400 mg	$62,171.69
valbenazine	11 Apr 2017	Other	Chronic Use (1 year)	Adult	28,800 mg	$81,591.72
vericiguat	19 Jan 2021	Other	Chronic Use (1 year)	Adult	3650 mg	$28,677.06
vibegron	23 Dec 2020	Other	Chronic Use (1 year)	Adult	27,000 mg	$5577.81
viloxazine	2 Apr 2021	Other	Chronic use (1 year)	Pediatric	67,200 mg	$7198.85
voclosporin	22 Jan 2021	Other	Chronic Use (1 year)	Adult	17,064 mg	$154,284.16
vosoritide	19 Nov 2021	Genetic Disorders	Chronic Use (1 year)	Pediatric	168 mg	$382,866.12
Orphan drugs New Biologics						
asparaginase erwinia chrysanthemi (recombi- nant)-rywn	30 Jun 2021	Oncology	Short treatment course	Adult & Pediatric	262.5 mg	$233,701.65
avalglucosidase alfa-ngpt	6 Aug 2021	Oncology	Chronic Use (1 year)	Adult & Pediatric	36,498 mg	$634,666.86
avelumab	23 Mar 2017		Cycles	Adult & Pediatric	18,200 mg	$149,490.89
belantamab mafodotin-blmf	5 Aug 2020	Oncology	Cycles	adult	3041.5 mg	$258,737.92
brexucabtagene autoleucel	24 Jul 2020	Oncology	Single-use	adult	1 × 10^6^ CAR-positive viable T cells/kg body weight	$460,217.51
burosumab-twza	17 Apr 2018	Genetic Disorders	Cycles	Adult & Pediatric	770 mg	$280,152.74
calaspargase pegol-mknl	20 Dec 2018	Oncology	Cycles	Adult & Pediatric	76,042 mg	$511,422.87
caplacizumab-yhdp	6 Feb 2019	Genetic Disorders	Short treatment course	Adult	330 mg	$230,772.87
cenegermin-bkbj	22 Aug 2018	Genetic Disorders	Short treatment course	adult	360 mg	$92,771.30
cerliponase alfa	27 Apr 2017	Genetic Disorders	Chronic Use (1 year)	Pedatric	7800 mg	$810,995.23
crizanlizumab-tmca	15 Nov 2019	Genetic Disorders	Cycles	Adult & Pediatric	4900 mg	$121,708.92
elapegademase-lvlr	5 Oct 2018	Genetic Disorders	Short treatment course	Adult & Pediatric	336 mg	$393,751.93
emapalumab-lzsg	20 Nov 2018	Genetic Disorders	Cycles	Adult & Pediatric	6720 mg	$230,768.11
emicizumab	16 Nov 2017	Genetic Disorders	Chronic Use (1 year)	Adult & Pediatric	4830 mg	$523,352.19
evinacumab-dgnb	11 Feb 2021	Genetic Disorders	Chronic Use (1 year)	Adult & Pediatric	12,600 mg	$399,262.34
ibalizumab-uiyk	6 Mar 2018	HIV	Chronic Use (1 year)	adult	21,120 mg	$128,579.61
inebilizumab-cdon	11 Jun 2020	Genetic Disorders	Cycles	adult	600 mg	$269,277.83
inotuzumab ozogamicin	17 Aug 2017	Oncology	Cycles	adult	11.4 mg	$258,152.31
lanadelumab	23 Aug 2018	Other	Cycles	Adult & Pediatric	7800 mg	$614,046.02
loncastuximab tesirine-lpyl	23 Apr 2021	Oncology	Cycles	adult	73.5 mg	$168,218.08
luspatercept-aamt	8 Nov 2019	Genetic Disorders	Cycles	Adult	1213 mg	$175,989.95
mogamulizumab-kpkc	8 Aug 2018	Genetic Disorders	Cycles	adult	1820 mg	$369,067.53
moxetumomab pasudotox-tdfk	13 Sep 2018	Oncology	Cycles	adult	50.4 mg	$114,368.37
naxitamab-gqgk	25 Nov 2020	Oncology	Cycles	Adult & Pediatric	3780 mg	$1,005,223.62
pegvaliase-pqpz	24 May 2018	Genetic Disorders	Chronic Use (1 year)	adult	1344 mg	$280,740.01
polatuzumab vedotin-piiq	10 Jun 2019	Oncology	Cycles	Adult	756 mg	$85,354.35
ravulizumab-cwvz	21 Dec 2018	Genetic Disorders	Chronic Use (1 year)	adult	21,450 mg	$470,793.46
ropeginterferon alfa-2b-njft	12 Nov 2021	Other	Chronic Use (1 year)	adult	2607 mg	$36,945.53
satralizumab-mwge	14 Aug 2020	Genetic Disorders	Chronic Use (1 year)	adult	1800 mg	$225,320.67
tafasitamab-cxix	31 Jul 2020	Oncology	Cycles	adult	12,618 mg	$77,811.02
tagraxofusp-erzs	21 Dec 2018	Oncology	Cycles	Adult & Pediatric	50,400 mg	$527,034.73
teprotumumab-trbw	21 Jan 2020	Other	Short treatment course	adult	10,500 mg	$321,591.73
vestronidase alfa-vjbk	15 Nov 2017	Genetic Disorders	Chronic Use (1 year)	Adult & Pediatric	2600 mg	$600,633.06
New Molecular Entities						
acalabrutinib	31 Oct 2017	Oncology	Cycles	adult	72,000 mg	$184,338.32
amifampridine phosphate	28 Nov 2018	Genetic Disorders	Chronic use (1 year)	adult	7200 mg	$131,934.10
asciminib	29 Oct 2021	Oncology	Chronic use (1 year)	adult	29,200 mg	$220,832.30
avacopan	7 Oct 2021	Other	Chronic use (1 year)	adult	21,900 mg	$178,244.98
avapritinib	9 Jan 2020	Oncology	Chronic use (1 year)	adult	109,500 mg	$400,148.23
belumosudil	16 Jul 2021	transplant	Cycles	Adult & Pediatric	11,560 mg	$30,281.42
belzutifan	13 Aug 2021	Genetic Disorders	Chronic use (1 year)	adult	43,800 mg	$325,696.80
benznidazole	29 Aug 2017	Infectious disease	Short treatment course	Pediatric	15,600 mg	$3604.42
berotralstat	3 Dec 2020	Genetic Disorders	Chronic use (1 year)	Adult & Pediatric	54,000 mg	$492,998.67
binimetinib	27 Jun 2018	Oncology	Chronic use (1 year)	adult	32,400 mg	$140,958.14
brigatinib	28 Apr 2017	Oncology	Chronic use (1 year)	adult	32,400 mg	$171,550.75
brilliant blue g	20 Dec 2019	Other	Single-Use	adult	0.5 mL	$236.60
cannabidiol	25 Jun 2018	Genetic Disorders	Chronic Use (1 year)	Pediatric	288,000 mg	$40,256.90
capmatinib	6 May 2020	Oncology	Chronic use (1 year)	adult	288,000 mg	$237,196.48
casimersen	25 Feb 2021	Genetic Disorders	Chronic use (1 year)	Pediatric	57,600 mg	$934,502.40
copanlisib	14 Sep 2017	Oncology	Cycles	adult	3120 mg	$238,549.30
copper Cu 64 dotatate injection	3 Sep 2020	Other	Single-Use	adult	4 mCi	$3597.22
dacomitinib	27 Sep 2018	Oncology	Chronic use (1 year)	adult	16,200 mg	$477,693.60
decitabine and cedazuridine	7 Jul 2020	Oncology	Cycles	adult	8100 mg	$92,438.35
deflazacort	9 Feb 2017	Genetic Disorders	Chronic use (1 year)	Pediatric	12,960 mg	$122,273.13
deutetrabenazine	3 Apr 2017	Genetic Disorders	Short treatment course	adult	1008 mg	$5484.97
duvelisib	24 Sep 2018	Oncology	Cycles	adult	16,800 mg	$151,526.48
edaravone	5 May 2017	Genetic Disorders	Cycles	adult	7200 mg	$1423.43
elexacaftor, tezacaftor, ivacaftor	21 Oct 2019	Genetic Disorders	Chronic use (1 year)	Adult & Pediatric	36,000 mg	$107,917.40
enasidenib	1 Aug 2017	Oncology	Chronic use (1 year)	adult	36,000 mg	$325,999.90
encorafenib	27 Jun 2018	Oncology	Chronic Use (1 year)	adult	162,000 mg	$140,958.14
entrectinib	15 Aug 2019	Oncology	Chronic use (1 year)	Adult & Pediatric	216,000 mg	$212,437.49
fedratinib	16 Aug 2019	Other	Cycles	Adult	144,000 mg	$265,546.86
fish oil triglycerides	27 Jul 2018	Other	Short treatment course	Pediatric	560 mg	$4563.54
fosdenopterin	26 Feb 2021	Genetic Disorders	Chronic use (1 year)	Pediatric	3285 mg	$480,314.73
fostamatinib	17 Apr 2018	Other	Chronic use (1 year)	adult	72,000 mg	$80,899.72
gilteritinib	28 Nov 2018	Oncology	Chronic use (1 year)	adult	43,200 mg	$288,927.58
givosiran	20 Nov 2019	Genetic Disorders	Chronic use (1 year)	adult	2100 mg	$456,627.56
glasdegib	21 Nov 2018	Oncology	Cycles	adult	33,600 mg	$202,848.56
golodirsen	12 Dec 2019	Genetic Disorders	Chronic use (1 year)	Pediatric	62,400 mg	$1,112,756.85
inclisiran	22 Dec 2021	Genetic Disorders	Chronic use (1 year)	adult	852 mg	$9903.94
infigratinib	28 May 2021	Oncology	Cycles	adult	31,500 mg	$81,753.72
inotersen	5 Oct 2018	Genetic Disorders	Chronic use (1 year)	adult	14,768 mg	$320,887.96
isatuximab	2 Mar 2020	Oncology	Cycles	adult	18,200 mg	$121,634.21
ivosidenib	20 Jul 2018	Oncology	Chronic use (1 year)	adult	180,000 mg	$347,577.05
larotrectinib	26 Nov 2018	Oncology	Chronic use (1 year)	Adult & Pediatric	67,200 mg	$421,192.21
letermovir	8 Nov 2017	transplant	Short treatment course	adult	48,000 mg	$21,299.04
lonafarnib	20 Nov 2020	Genetic Disorders	Chronic use (1 year)	Pediatric	40,800 mg	$1,272,021.04
lonapegsomatropin-tcgd	25 Aug 2021	Other	Chronic use (1 year)	Pediatric	473.2 mg	$24,194.24
lorlatinib	2 Nov 2018	Oncology	Chronic use (1 year)	adult	36,000 mg	$206,176.28
lumasiran	23 Nov 2020	Genetic Disorders	Cycles	Adult & Pediatric	450 mg	$538,575.32
lurbinectedin	15 Jun 2020	Oncology	Cycles	adult	97.3 mg	$165,886.45
lutetium Lu 177 dotatate	26 Jan 2018	Oncology	Cycles	adult	800 mg	$109,902.38
macimorelin acetate	20 Dec 2017	Other	Single-Use	adult	35 mg	$2527.44
maralixibat	29 Sep 2021	Genetic Disorders	Chronic use (1 year)	Pediatric	2357.9 mg	$390,095.94
maribavir	23 Nov 2021	infectious disease	Short treatment course	adult	48,000 mg	$54,103.80
melphalan flufenamide	26 Feb 2021	Oncology	Cycles	Adult	160 mg	$77,064.00
midostaurin	28 Apr 2017	Oncology	Cycles	adult	42,000 mg	$245,594.36
migalastat	10 Aug 2018	Genetic Disorders	Chronic use (1 year)	adult	20,664 mg	$259,499.77
mobocertinib	15 Sep 2021	Oncology	Chronic use (1 year)	adult	53,760 mg	$283,920.00
nifurtimox	6 Aug 2020	Infectious disease	Short treatment course	Pediatric	180 mg	$587.01
niraparib	27 Mar 2017	Oncology	Chronic use (1 year)	adult	108,000 mg	$253,639.89
odevixibat	20 Jul 2021	Genetic Disorders	Chronic use (1 year)	Pediatric	268,800 mg	$299,819.52
osilodrostat	6 Mar 2020	Other	Chronic use (1 year)	adult	5110 mg	$420,155.64
patisiran	10 Aug 2018	Genetic Disorders	Cycles	adult	364 mg	$370,041.32
pegcetacoplan	14 May 2021	Genetic Disorders	Chronic use (1 year)	adult	103,680 mg	$428,687.40
pemigatinib	17 Apr 2020	Oncology	Cycles	adult	2268 mg	$629,000.12
pexidartinib	2 Aug 2019	Oncology	Cycles	Adult	28,8000 mg	$250,372.75
pitolisant	14 Aug 2019	Other	Chronic use (1 year)	Adult	12,816 mg	$143,774.66
pralsetinib	4 Sep 2020	Oncology	Chronic use (1 year)	adult	144,000 mg	$237,332.85
ripretinib	15 May 2020	Oncology	Chronic use (1 year)	adult	54,000 mg	$473,789.61
risdiplam	7 Aug 2020	Genetic Disorders	Chronic use (1 year)	Pediatric	1800 mg	$339,668.66
selpercatinib	8 May 2020	Oncology	Chronic use (1 year)	Adult & Pediatric	115,200 mg	$254,066.72
selumetinib	10 Apr 2020	Genetic Disorders	Cycles	Adult & Pediatric	32,400 mg	$242,091.05
setmelanotide	25 Nov 2020	Other	Chronic use (1 year)	Adult & Pediatric	720 mg	$244,200.05
solriamfetol	20 Mar 2019	Other	Chronic use (1 year)	Adult	27,000 mg	$4172.88
sotorasib	28 May 2021	Oncology	Chronic use (1 year)	adult	292,032 mg	$180,003.02
stiripentol	20 Aug 2018	Genetic Disorders	Chronic Use (1 year)	Pediatric	720,000 mg	$81,491.71
tafamidis meglumine	3 May 2019	Other	Chronic use (1 year)	Adult	28,800 mg	$237,095.41
tafenoquine	20 Jul 2018	Infectious disease	Single-Use	adult	300 mg	$34.24
tucatinib	17 Apr 2020	Oncology	Chronic use (1 year)	adult	216,000 mg	$228,166.71
umbralisib	5 Feb 2021	Oncology	Chronic use (1 year)	adult	292,000 mg	$196,158.30
viltolarsen	12 Aug 2020	Genetic Disorders	Chronic use (1 year)	Adult & Pediatric	268,800 mg	$544,028.87
voxelotor	25 Nov 2019	Genetic Disorders	Chronic use (1 year)	Adult & Pediatric	540,000 mg	$131,723.88
zanubrutinib	14 Nov 2019	Oncology	Chronic use (1 year)	Adult	115,200 mg	$163,564.22

mCi = millicurie (radioactivity units).

**Table A2:** Correlation matrix.

	Treatment Cost of New Drugs at Market Entry	Date of Market Entry	Application Type	Orphan	Priority Review	First in Class Drugs	Country of Incorporation	Therapeutic Intent	Treatment Duration	Therapeutic Area	Age Group
Treatment Cost of New Drugs at Market Entry	1	0.023	0.28	0.548	0.378	0.21	−0.14	0.452	0.452	0.452	0.452
Date of Market Entry		1	0.045	−0.007	0.116	−0.146	−0.036	0.852	0.08	0.83	0.535
Application Type			1	0.05	0.007	0.186	0.198	0.103	0.133	0.238	0.179
Orphan				1	0.381	0.202	0.053	0.079	0.154	0.623	0.159
Priority Review					1	0.307	0.122	0.098	0.226	0.582	0.159
First in Class Drugs						1	0.008	0.065	0.053	0.277	0.1
Country of Incorporation							1	0.065	0.047	0.113	0.102
Therapeutic Intent								1	0.428	0.186	0.077
Treatment Duration									1	0.351	0.108
Therapeutic Area										1	0.332
Age Group											1

Cramer’s V, point-biserial, Kruskal. Test and Spearman correlation coefficients were used.

**Table A3:** Multicollinearity.

Independent Variable	VIF	Increased SE	Tolerance
Year of Market Entry	1.15	1.07	0.87
Application Type	1.24	1.11	0.81
Orphan	1.78	1.33	0.56
Country of Incorporation	1.08	1.04	0.92
Priority.Review	1.84	1.35	0.54
First in Class Drugs	1.34	1.16	0.75
Intent	1.81	1.34	0.55
Therapeutic Area	4.23	2.06	0.24
Age Group	1.47	1.21	0.68
Treatment Duration	2.24	1.5	0.45

Variance inflation factor (VIF), standard error (SE). We checked the multicollinearity of independent variables by using VIF for each independent variable in the set of multiple regression variables. The higher the value of VIF, the higher the correlation between this variable and the rest. If the VIF value is higher than 5, it is usually considered to have a high correlation with other independent variables. However, the value of VIF for each independent variable included in our model was less than 5. Our data show low multicollinearity; it is not severe enough to warrant corrective measures.
